# Expanding the global diversity of *Nanobdellales* from deep-sea hydrothermal vents

**DOI:** 10.1093/ismeco/ycag280

**Published:** 2026-10-01

**Authors:** Anna-Louise Reysenbach, Emily St. John, Ryan Catchpole

**Affiliations:** Center for Life in Extreme Environments, Portland State University, Portland, OR 97201, United States; Center for Life in Extreme Environments, Portland State University, Portland, OR 97201, United States; Department of Microbiology, University of Georgia, Athens, GA 30602, United States

**Keywords:** deep-sea hydrothermal vent, symbiosis, DPANN, *Nanobdellota*, thermophile

## Abstract

The archaeal symbiotic *Nanobdellales* have been isolated with their hosts from a shallow hot marine vent off Iceland and from terrestrial hot springs in Yellowstone National Park and Japan. They have highly reduced genomes and rely on their archaeal hosts for most of their metabolic needs. Here, to provide further insights into the ecology and evolution of this order, we greatly expand their phylogenomic distribution using metagenome-assembled genomes from deep-sea hydrothermal vents in the Atlantic, Pacific and Indian Oceans, and the Gulf of California. We show that at least 11 novel genus-level lineages occur at deep-sea vents. We conservatively propose two new genera with their nomenclatural types, *Nanoprofundum liuii* Rb-1^Ts^ gen. nov. sp. nov. and *Nanojejuna irisiana* Rb-38^Ts^ gen. nov. sp. nov. We also describe a second species of *Nanoarchaeum*, *N. abyssimaris* 17S-22^Ts^ sp. nov. Our genomic and metabolic characterization of the high-quality metagenome-assembled genomes confirm their highly reduced metabolic capabilities and likely symbiotic lifestyle. With the exception of *Nanoarchaeum,* all deep-sea genera resemble their terrestrial relatives in that they have genes that encode for archaella and RNAase P protein subunits, and have several genes involved in glycolysis and gluconeogenesis. Using an inferred lateral gene transfer analysis, our data suggest that deep-sea *Nanobdellales* may potentially associate with a greater taxonomic breadth of hosts than previously reported, spanning members of the *Sulfolobales* (*Ignicoccaceae*), the *Archaeoglobales* and *Methanococcales*.

## Introduction

Within the *Archaea*, the kingdom *Nanobdellati* (previously the DPANN superphylum) [1, 2] is a diverse group comprising at least ten phyla whose nomenclature has recently been revised [3]. These include the *Iainarchaeota*, *Microcaldota*, *Aenigmatarchaeota*, *Undinarchaeota, Terrarchaeota, Huberarchaeota, Altarchaeota, Nanobdellota,* and *Nanohalarchaeota*. They have a widespread distribution from saline lakes, hot geothermal springs, sediments, freshwater and marine environments, and the deep subsurface [4, references therein]. Most have reduced, small genomes. Although members of the *Altarchaeota* are predicted to be free-living [5, 6], all cultivated *Nanobdellati* are symbiotic with other microorganisms [7–16]. They are likely important ecologically, based on their distribution and interactions with their hosts and their community.

The first *Nanobdellati* grown in co-culture with its host [7] was a member of the *Nanobdellales*. Subsequently, several more novel genera have been cultivated from this order. These co-cultures include *Nanoarchaeum* in the *Nanoarchaeaceae* and *Nanopusillus*, *Nanobdella, Nanoclepta,* and *Candidatus* (*Ca.*) Nanofervidus in the *Nanobdellaceae*, whose hosts are *Ignicoccus*, *Acidilobus*, *Metallosphaera, Zestosphaera,* and *Sulfurisphaera*, respectively [8–11]. While the symbionts were isolated with specific hosts, there is evidence that some can form associations with different strains of the same species [11].

All *Nanobdellales* have several features in common. They have reduced genomes (~0.491 to 0.773 Mbp) and lack the ability to synthesize amino acids, co-factors, lipids, and nucleotides [8–11, 17]. However, they have retained genes involved in replication, transcription, and translation. Furthermore, unlike *Nanoarchaeum equitans* [17, 18]*,* the terrestrial hot spring-associated *Nanobdellales* have some genes that encode for glycolysis, gluconeogenesis, and archaella, but do not encode genes for a partial ATP synthase [8–11].

The *Nanobdellales*-host cultures all were isolated from high temperature terrestrial hot springs, except for *Nanoarchaeum*, which was obtained from a shallow marine hot spring off the coast of Iceland [7]. However, *Nanobdellales* have been detected at deep-sea vents through 16S rRNA gene amplicon sequencing [19], cloning [20], and recently from metagenomes [21, 22], and have been implicated as early colonizers of hydrothermal deposits [20].

We previously recovered three *Nanobdellales* metagenome-assembled genomes (MAGs) from samples collected at three deep-sea hydrothermal vent fields in the Pacific, Gulf of California, and Caribbean. Consistent with other *Nanobdellales* shared features, including reduced biosynthetic and energy generation capabilities, these initial deep-sea *Nanobdellales* genomes most closely resembled their terrestrial relatives such as having marker genes for gluconeogenesis and archaella, and lacking any ATP synthase genes [22].

To further explore the diversity of deep-sea *Nanobdellales*, we have screened 112 publicly available deep-sea hydrothermal vent-associated metagenomes from a total of 23 different locations along mid-ocean ridges, back-arc spreading centres, and volcanic arcs [21, 23, 24] for the presence of *Nanobdellales*. We identified 31 *Nanobdellales* MAGs that represent about 11 unnamed genera and confirm that MAGs of most deep-sea *Nanobdellales* are more similar to their terrestrial relatives than the marine *Nanoarchaeum equitans.* Further, we propose two new genera based on type MAGs with high-quality genomes and representation from multiple deep-sea vent metagenomes.

## Materials and methods

### Dataset descriptions

Datasets used in this study included phyloFlash analysis [25] from the DSV70 deep-sea vent metagenomes [23], raw metagenomic reads from the Eastern Lau Spreading Center-Valu Fa Ridge (2015) [21] and Brothers deep-sea volcano (2018) [21, 24], and trimmed deep-sea vent metagenome reads sequenced at the US Joint Genome Institute (JGI) from the Mid-Atlantic Ridge, Eastern Lau Spreading Center-Valu Fa Ridge, Guaymas Basin, and East Pacific Rise [21]. Deep sea vent-associated MAGs were recovered from the DSV70 dataset [23] and studies described by Zhou *et al.* [21] and St. John *et al.* [22].

### PhyloFlash analysis of metagenomes

Raw reads described above were trimmed with the same protocol used for DSV70 [23]. Trimmed reads (42 datasets in all) were analysed with phyloFlash as described by St. John & Reysenbach [23] (Table S1). Small subunit rRNA genes were also assembled from each trimmed read set using phyloFlash when possible.

### Identification and genomic analysis of *Nanobdellales* MAGs

MAGs assigned to the *Nanobdellales* in the DSV70 dataset were initially taxonomically assigned and placed in a phylogenomic tree with GTDB-Tk v2.4.1, database R10-226 [26, 27]. MAGs were manually curated using taxonomic classification of contigs with the CAT/BAT tool [28]. Two previously published MAGs [22], M10-121 (Genbank assembly accession GCA_004285895.1) and Gua-46 (GCA_004285905.1) were also curated with CAT/BAT. Genbank records were updated to reflect the curated sequences. Additional publicly available reference genomes, and when available, predicted proteins and ribosomal RNA genes were downloaded from NCBI Genbank (Table S2, S3) [8–11, 17, 29, 30]. Completeness and contamination were estimated using CheckM2 v1.1.0 with the parameter --allmodels [31]. Proteins and rRNA genes of the deep-sea MAGs and the hot-spring-associated genome CTOTU25739 (GCA_036845085.1) were predicted with Prodigal v2.6 [32] and barrnap v0.9 (https://github.com/tseemann/barrnap) via the Prokka v1.13 pipeline [33]. Transfer RNA genes were predicted separately using the tRNAscan-SE v2.0.12 command line tool with the archaeal-specific option -A [34] and were compared to previously reported counts when applicable (Table S3) [8–11, 35]. CRISPR repeat regions were predicted using the command-line CRISPRCasFinder v4.3.2 tool [36] with the parameter --keep, and only those with evidence level four were considered [36]. CRISPR spacer regions were screened against the viral NCBI core nucleotide database (taxid 10 239) using the blastn web interface with an E-value cutoff of 1E-5 [37–39]. Average amino acid identity (AAI) analysis was done using the comparem v0.1.2 aai_wf module, with the setting --blastp (https://github.com/donovan-h-parks/CompareM; Table S4), and average nucleotide identity (ANI) comparisons were accomplished using pyani v0.3.0-alpha with the setting -m ANIb (Table S5) [40]. AAI values were visualized as a heatmap in Morpheus (https://software.broadinstitute.org/morpheus/), using one minus Pearson correlation with average linkage for hierarchical clustering.

### Phylogeny and 16S rRNA gene analysis

Pairwise comparisons of 16S rRNA genes were done in EZBioCloud [41]. The 16S rRNA gene tree was constructed from near full-length sequences derived from reference genomes, MAGs, and phyloFlash reconstruction using the SILVA ACT web server [42]. Sequences were aligned with SINA v1.2.12 via the global SILVA alignment with the archaeal variability profile [43]. A maximum-likelihood tree was inferred by SILVA ACT using RAxML under the GTR + Gamma model, with the archaeal domain setting and a positional variability filter excluding highly and moderately variable positions [42, 44]. Rapid bootstrapping (1000 replicates) was performed with RAxML v8.2.12 [44]. A second tree was generated using the same workflow with 16S rRNA gene fragments from MAGs and phyloFlash reconstruction, and two partial 16S rRNA genes from the M10 and S143 assemblies [21] obtained with barrnap v0.9 and initially identified with the RDP Classifier v2.14 [45]. Phylogenomic analysis was done with an alignment of 53 archaeal marker genes using FastTree 2 v2.2.0 (parameter --gamma) [46] via GTDB-Tk v2.4.1, database R10-226 [26] with the default archaeal database. Local branch support values were calculated in FastTree 2 using 1000 resamples of the Shimodaira-Hasegawa test [47]. All trees were visualized using Interactive Tree of Life v7 [48]. Where applicable, novel taxon names were formulated in accordance with the recommendations of the SeqCode [49].

### Functional predictions

Annotation for MAGs and reference genomes was performed using the GhostKoala web server [50]. Blastp searches (E-value cutoff 1E-5) [38] were done against the Archaeal Clusters of Orthologous Genes (arCOG) database version ar18 [51], with the best hit for each predicted protein identified using the best_blast.pl script (https://github.com/jmeneghin/perl-for-reysenbach-lab/blob/master/blast_best.pl; Table S6, S7, S8). The Conserved Domain (CD) Database batch search tool [52] was used to verify conflicting annotations as needed, and to verify putative form III RuBisCO hits. A heatmap of predicted functional gene patterns was generated with Morpheus.

### Putative lateral gene transfer analysis

Predicted proteins from all representative genomes in the GTDB database R10-226 [27] were downloaded and a database was generated from all protein sequences using DIAMOND v2.1.21.175 [53]. To identify potential lateral gene transfer (LGT) events, predicted proteins from deep-sea vent *Nanobdellales* MAGs were screened against the database using a DIAMOND blastp search with an E-value cutoff of 1E-5 [38, 53]. Best matches were identified with the blast_best.pl script (see “Functional predictions”) (Table S9), assigned to GTDB taxonomic categories and assessed at the order level. Orders outside the *Nanobdellati* with at least three putative hits in any single *Nanobdellales* genome were considered for analysis (Table S10). To rule out false positives in the database, we identified contigs from orders of interest with at least two hits in a single MAG. These contigs were screened with the CAT/BAT contig annotation tool [28], leading to the identification of four putative *Nanobdellales* contigs assigned to MAGs in other taxonomic groups. All hits to these contigs were removed from the analysis (Table S9). The results were visualized as a heatmap using Morpheus.

### Map generation

A *Nanobdellales* detection map was generated using arGIS Online with the General Bathymetric Chart of the Oceans (GEBCO) Basemap (NOAA NCEI Visualization) and the Esri Oceans Plate Boundaries layer [54]. Published coordinates were used for the 112 metagenomes screened for *Nanobdellales* [21, 23], cultivated *Nanobdellales-*host systems [8–10, 55, 56], and the MC-1 deep-sea vent MAG [57].

## Results and discussion

### Diversity and phylogeny of deep-sea hydrothermal vent *Nanobdellales* MAGs

*Nanobdellales* 16S rRNA gene fragments were detected in 86 of 112 publicly available deep-sea hydrothermal vent-associated metagenomes [21, 23]. However, the low number of *Nanobdellales* reads limited MAG reconstruction, with only 37 of these metagenomes having at least 20 reads that mapped back to *Nanobdellales* 16S rRNA genes (Fig. 1, Table S1). After MAG assembly, only 17 of the 37 metagenomes contained one or more medium- to high-quality *Nanobdellales* MAGs (≥50% completion, <10% contamination, Table 1, Tables S2, S3). A total of 31 *Nanobdellales* MAGs were obtained that included the five MAGs previously reported [21, 22]. A third of these (ten) were obtained from Guaymas Basin metagenomes (Fig. 2). Based on phylogenomic analysis (Fig. 2) and AAI analysis (Fig. S1, Table S4) [58] the new *Nanobdellales* MAGs reported here represent at least 11 novel genus-level lineages, greatly expanding the known phylogenetic diversity of the order. Five of these lineages are only represented by a single MAG and therefore are not considered in detail here.

**Figure 1:**
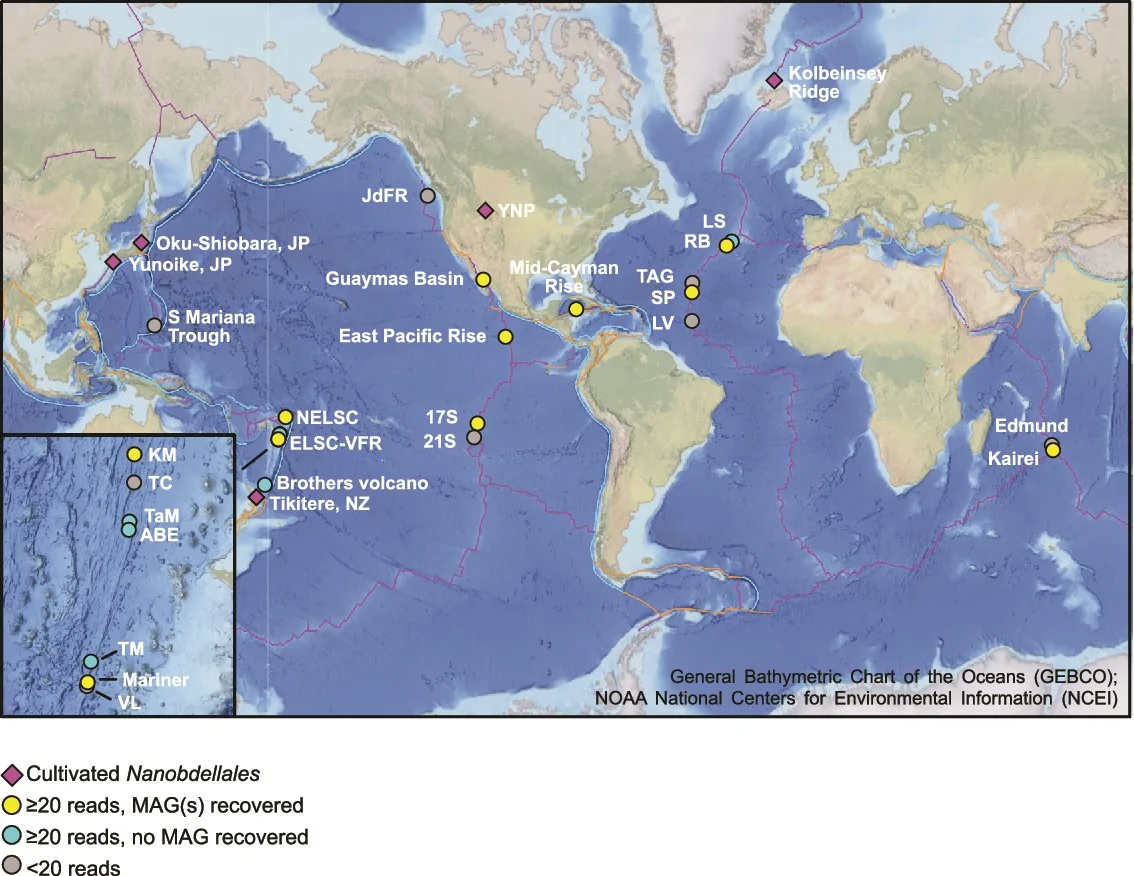
Sites where *Nanobdellales* were recovered from the metagenomes of deep-sea vent samples. Geographic locations are coloured according to recovery of MAG(s) and number of reads mapped to *Nanobdellales* 16S rRNA genes by phyloFlash (SILVA categories *Nanoarchaeaceae, Nanopusillaceae,* and unclassified *Nanoarchaeales*). The inset shows locations along the Eastern Lau Spreading Center-Valu Fa Ridge (ELSC-VFR). JdFR, Juan de Fuca Ridge; JP, Japan; KM, Kilo Moana; LV, Logatchev; LS, Lucky Strike; NELSC, Northeastern Lau Spreading Center; NZ, New Zealand; RB, Rainbow; SP, Snake Pit; TaM, Tahi Moana; TC, Tow Cam; TM, Tui Malila; USA, United States of America; VL, Vai Lili; YNP, Yellowstone National Park.

**Table 1:** Genome features of published *Nanobdellales* reference genomes and deep-sea vent MAGs. For each clade (see Fig. 2), MAGs with the highest estimated completion under the CheckM2 general model are shown. See Tables S2 and S3 for full characteristics of all MAGs and reference genomes utilized.

MAG	Clade	Assembly accession	Site	Comp (%)	Con (%)	Genome size (bp)	GC (%)	Contigs (#)	Prot (#)	16S rRNA gene (bp)
*Nanoarchaeum equitans* Kin4-M^Ts^	A	GCA_000008085.1	Kolbeinsey Ridge	96.85	0.41	490 885	31.56	1	536	1500
*Nanoclepta minutus* Ncl-1^Ts^	H	GCA_003568775.1	Tikitere, NZ	91.22	0.68	575 637	32.19	9	644	1502
*Nanobdella aerobiophila* MJ1^T^	-	GCA_023169545.1	Oku-Shiobara, JP	96.28	0.56	668 961	24.64	1	736	1440
*Ca*. Nanofervidus parviconus YN1	-	GCA_042850255.1	Yunoike, JP	95.27	0.86	773 326	23.37	1	889	1497
*Nanopusillus acidilobi* 7A^Ts^	-	GCA_001552015.1	YNP, USA	95.35	0.55	605 887	24.24	1	587	1488
***Nanoarchaeum abyssimaris* 17S-22** ^**Ts**^	A	GCA_054016815.1	17° 25’ S, SEPR	96.59	1.04	637 833	36.36	23	723	1497
3327-2_bin.8	B	GCA_056892115.1	17° 25’ S, SEPR	88.31	0.72	640 590	30.6	77	796	—
MS15-CHA3_bin.7	B	GCA_054017035.1	Snake Pit, MAR	94.84	2.96	648 387	30.09	22	812	—
3517-1-1_bin.14	C	GCA_056892095.1	Guaymas Basin	90.23	2.63	689 609	31.01	55	808	—
3517-1-1_bin.183	C	GCA_056892015.1	Guaymas Basin	91.54	6.8	686 324	32.52	41	760	509
3517-1-1_bin.143	S	GCA_053994055.1	Guaymas Basin	90.86	0.26	641 398	36.15	33	743	551
***Nanoprofundum liuii* Rb-1** ^**Ts**^	D	GCA_056891935.1	Rainbow, MAR	94.79	0.24	540 544	23.69	39	573	1500
355–1-R3–1-177_bin.60	D	GCA_056891895.1	Rainbow, MAR	91.99	0.29	502 194	23.57	35	542	1500
***Nanojejuna irisiana* Rb-38** ^**Ts**^	E	GCA_056891915.1	Rainbow, MAR	91.08	0.83	613 650	32.12	28	699	1489
355–1-R3–1-177_bin.9	E	GCA_056891815.1	Rainbow, MAR	88.52	0.26	543 675	32.34	79	617	1244
124–5-R1–1-33_bin.76	E	GCA_056891365.1	Kilo Moana, ELSC-VFR	87.63	0.86	616 090	28.92	41	689	1481
416–21-R1–1-10_bin.67	S	GCA_056891785.1	Maka, NELSC	93.86	0.68	737 933	28.42	5	811	404
416–21-R1–1-10_bin.36	S	GCA_056891735.1	Maka, NELSC	93.66	0.33	616 283	30.11	15	698	404
MS15-CHA3_bin.28	F	GCA_056891305.1	Snake Pit, MAR	90.39	0.33	624 038	30.23	34	729	757
MC-1	F	GCA_020697515.1	Mid-Cayman Rise	84.66	0.24	469 257	32.21	55	499	1517
4559–3-258_bin.313	G	GCA_056891465.1	Guaymas Basin	83.68	1.57	521 119	35.28	88	593	1478
Adv9-388_bin.54	G	GCA_056891445.1	9ºN, EPR	89.65	0.73	679 999	30.41	31	772	515
124–5-R1–1-33_bin.15	H	GCA_056892175.1	Kilo Moana, ELSC-VFR	90.95	0.68	544 244	31.39	21	617	—

CheckM2 was used to estimate completeness and contamination, and % G + C content was determined with the get_gc_by_bin.pl script (https://github.com/ALR-Lab/get_gc_by_bin.pl). Clade designations are shown in Fig. 2, and singletons are marked as “S.” Novel taxa proposed under the SeqCode are shown with the superscript “Ts” and the ICNP validated taxon is shown with the superscript “T.” NZ = New Zealand; JP = Japan; YNP = Yellowstone National Park; USA = United States of America; SEPR = Southern East Pacific Rise; MAR = Mid-Atlantic Ridge; ELSC-VFR = Eastern Lau Spreading Center-Valu Fa Ridge; NELSC = Northeastern Lau Spreading Center; EPR = East Pacific Rise; Comp (%) = percent estimated completeness under the CheckM2 general model; Con (%) = percent estimated contamination; GC (%) = G + C content as a percentage; Contigs (#) = number of contigs in each MAG; Prot (#) = number of predicted proteins per MAG; 16S rRNA gene (bp) = length of predicted 16S rRNA gene(s) in base pairs.

**Figure 2:**
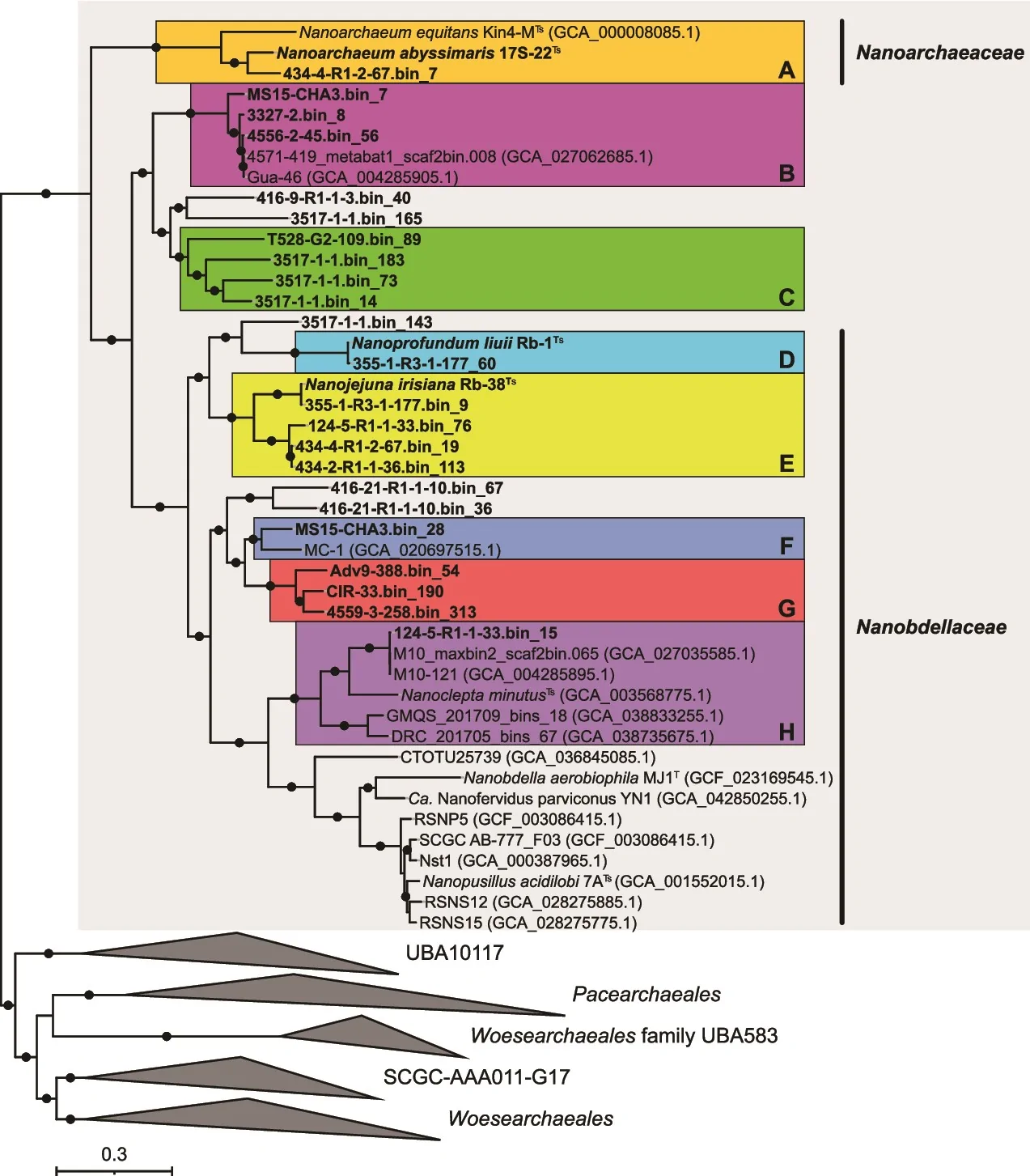
Phylogenomic placement of deep-sea hydrothermal vent-associated genera in the *Nanobdellales* (grey box)*.* Closely related lineages are shown as collapsed wedges, labelled per Genome Taxonomy Database (GTDB) database release R10–226 nomenclature. Deep-sea associated genera with at least two MAGs are indicated with coloured boxes, and MAGs from the DSV70 dataset are shown in bold. Type sequences proposed under the SeqCode are indicated with the superscript “Ts” and the type strain validly published under the ICNP is shown with a superscript “T.” Genbank assembly accession numbers for reference genomes and previously published MAGs are shown in parentheses. The scale bar represents 0.3 amino acid substitutions per site, and local support values from 0.8 to 1 are shown with filled dots. The complete phylogenomic tree used to construct this figure is publicly available on iTOL at https://itol.embl.de/shared/alrlab.

Two *Nanoarchaeum* MAGs were obtained from the Southern East Pacific Rise (SEPR) and the Kilo Moana vent field along the Eastern Lau Spreading Center-Valu Fa Ridge (ELSC-VFR) (Fig. 1, Fig. 2, Clade A); and ANI analysis suggests that both MAGs represent distinct *Nanoarchaeum* species (Table S5). Since a near-complete 16S rRNA gene (1497 bp) from MAG 17S-22 was recovered that was 96.39% similar to the *Nanoarchaeum equitans* 16S RNA gene (Fig. S2), and based on genome features described fully below, we propose naming this new MAG *Nanoarchaeum abyssimaris* 17S-22^Ts^ sp. nov. Likewise, based on genome completeness, coverage, and presence of a 16S rRNA gene, and the taxonomic congruence between phylogenomic analysis (Fig. 2), 16S rRNA phylogeny (Fig. S2) and AAI analysis (Fig. S1), we propose naming two new genera within the *Nanobdellales, Nanoprofundum* gen. nov. (Fig. 2, Clade D) and *Nanojejuna* gen. nov. (Fig. 2, Clade E), with *Nanoprofundum liuii* Rb-1^Ts^ sp. nov. and *Nanojejuna irisiana* Rb-38^Ts^ sp. nov. as their respective nomenclatural types. Although GTDB taxonomic analysis did not put these clades in a known family, we have chosen to conservatively place them in the *Nanobdellaceae* based on branching patterns in phylogenomic analysis (Fig. 2), the 16S rRNA gene tree (Fig. S2) and AAI analysis (Fig. S1, Table S4).

Several of the novel genus-level clades appear to be geographically widespread and temporally persistent, with representative MAGs recovered from multiple deep-sea hydrothermal sites across oceans or over multiple collection years (Table S3). For example, Clade B is represented by three species from the Snake Pit hydrothermal vent site along the Mid-Atlantic Ridge (MAR), from vents on the SEPR and in Guaymas Basin in the Gulf of California. Likewise, Clade G has members that are from the East Pacific Rise (EPR), the Kairei vent field along the Central Indian Ridge (CIR), and Guaymas Basin. MAGs related to the proposed *Nanojejuna irisiana* from the MAR Rainbow deep-sea vents were assembled from samples collected during two different expeditions to Kilo Moana along the ELSC-VFR in 2005 and again in 2009. Kilo Moana was also the source of another MAG related to *Nanoclepta*, the *Nanobdellales* cultivated from a New Zealand hot spring [9]. Another MAG related to this group was previously reported from the Mariner vent field along the ELSC-VFR [22]. Although we were unable to identify a MAG from Brothers volcano, a partial (904 bp) 16S rRNA gene recovered from the S143 metagenome assembly clustered with *Nanoclepta minutus,* suggesting a broader geographic distribution of *Nanoclepta-*related taxa (Fig. 1, Fig. S3). In contrast, the only MAGs obtained from the Northeastern Lau Spreading Center (NELSC) deep-sea vent field were three of the five lineages that have singleton MAGs in our study. This may point to potential high endemism of new *Nanobdellales* genera at this site.

### Genomic features of deep-sea hydrothermal vent MAGs

Consistent with the reduced genomes of *Nanobdellales* in co-culture with their hosts, the genome size of our high-quality type MAGs ranged from 540.5 (*Nanoprofundum liuii*) to 637.8 kbp (*Nanoarchaeum abyssimaris*). Because of their reduced genome size, calculating completion in symbionts can be problematic. However, using the general model of CheckM2 [31], completion estimates for our type MAGs (91.08% to 96.59%) were close to those calculated for known complete genomes of isolates (95.27% to 96.85%; Table S3). Further, the number of predicted proteins was similar to those reported for cultivated *Nanobdellales*.

All the MAGs shared core features of the *Nanobdellales*, such as encoding genes for transcription, translation, and DNA replication, accounting for an average of ~35% of the predicted proteins in each MAG (arCOG categories J, K, L; Tables S6, S7, S8), and lacking genes for central metabolism, including the pentose phosphate pathway, tricarboxylic acid (TCA) cycle, respiration and biosynthesis of vitamins, amino acids, lipids, and cofactors. Further, like published co-cultures of *Nanobdellales*, the MAGs contain major pilin and S-layer genes (Fig. 3). CRISPR repeats were also identified in several deep-sea lineages including *Nanoarchaeum, Nanoprofundum,* and *Nanojejuna.* Like *N. equitans* [59]*, N. abyssimaris* and *Np. liuii* encoded for most of the Type I *cas* genes (Table S7). No putative viral targets were identified by screening with the NCBI core_nt viral database [39].

**Figure 3:**
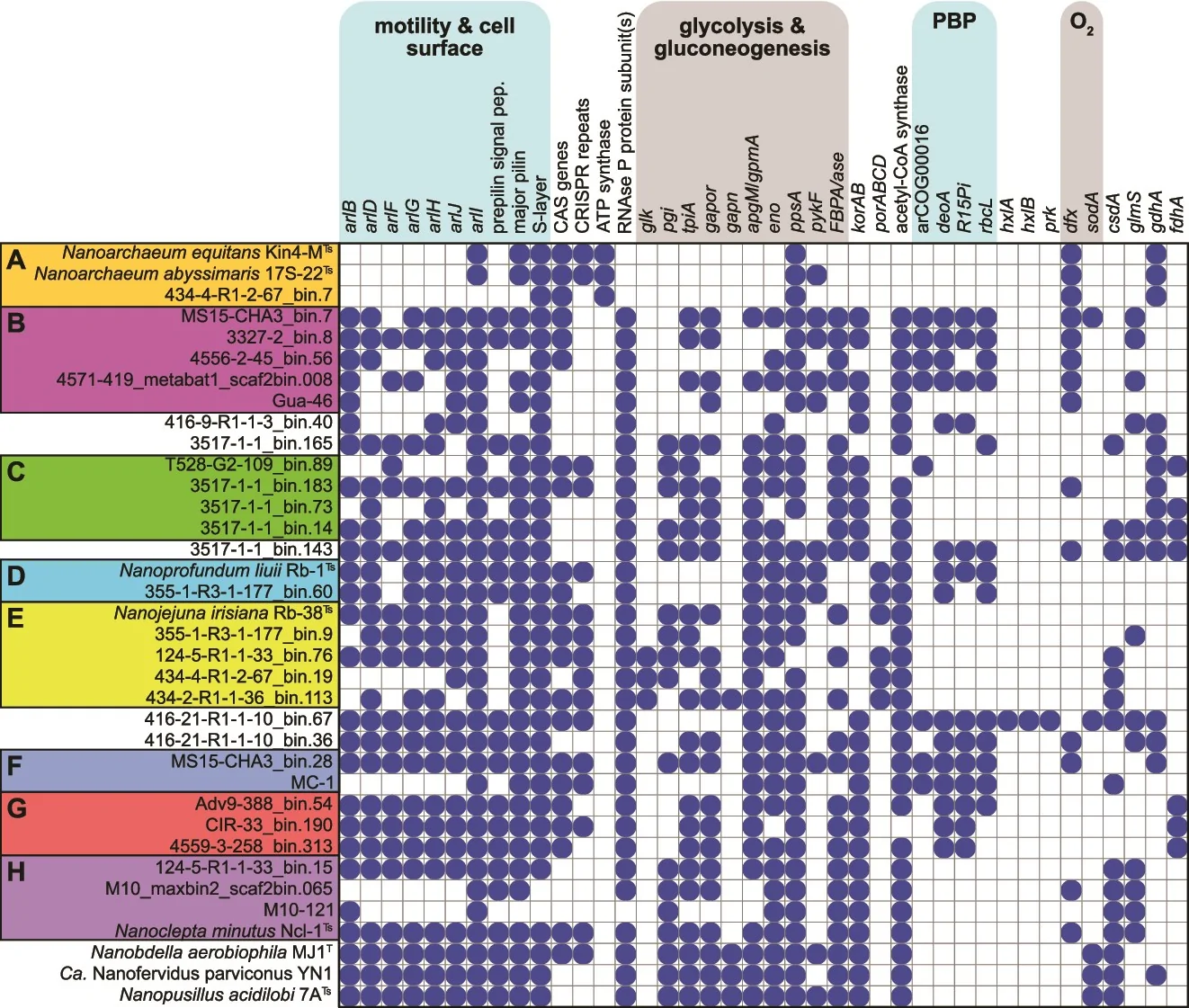
Comparative functional analysis for deep-sea vent-associated *Nanobdellales* MAGs and reference genomes. Putative genera correspond to Fig. 2. Filled circles depict identified genes and CRISPR repeat regions. Full gene descriptions are available in Table S7. PBP, pentose bisphosphate pathway; O_2_, oxygen tolerance.

However, our study further highlights that the genus *Nanoarchaeum* is more the exception than the rule in the *Nanobdellales*. For example, there is no evidence that *Nanoarchaeum* encodes genes for archaellum formation (*arlBDFGHJ*; Fig. 3), yet all other co-cultured genera appear to have the potential to produce archaella or have been shown to be motile [8–11, 60]. Although some archaella genes (*arlDFG*) were undetected in the initial *N. aerobiophila* genome analysis [10], we were able to identify putative homologues for each of these genes using the arCOG database [51] (Tables S7, S8). Additionally, the genes encoding a non-functional ATP synthase in *N. equitans* [17, 18] were also present in our deep-sea *Nanarchaeum* MAGs but absent from all other *Nanobdellales.* Further, genes encoding protein subunits of RNAase P were not detected in *Nanoarchaeum* [61] but were detected in all other genera of the *Nanobdellales* (Fig. 3).

Like their terrestrial relatives, most of the MAGs encoded several genes involved in glycolysis and gluconeogenesis, except for the *Nanoarchaeum* lineage where these genes are absent. However, even when present, the pathways appear incomplete. For example, in glycolysis, no genes for converting fructose 6-phosphate to fructose 1,6-bisphosphate to glyceraldehyde 3-phosphate (GAP) were detected (Table S7). Likewise in gluconeogenesis, genes for conversion of 3-phosphoglycerate to GAP were not detected in the MAGs, although most encode a putatively reversible ferredoxin-dependent aldehyde oxidoreductase (*gapor*) that may function in the anabolic direction [8]. Two key genes in gluconeogenesis [62], bifunctional fructose-1,6-bisphosphate aldolase/phosphatase (*FBPA/ase*) and phosphoenolpyruvate synthase (*ppsA*), were also identified in most MAGs (Fig. 3).

Genes involved in the pentose bisphosphate pathway (PBP) used to degrade nucleoside monophosphates in Archaea [63–65] were previously identified in a *Nanobdellales* MAG from the Mid-Cayman Rise deep-sea vents [22]. Here, we identified PBP genes including the archaeal type-III RuBisCO in the *Nanoprofundum* and clades B, F, and G (Fig. 3). It has been proposed previously that, if functional, the 3-phosphoglycerate produced by nucleoside degradation via the PBP may enter the lower portion of glycolysis and subsequently produce ATP [66].

Notably, a few other predicted proteins previously not reported in the *Nanodbellales* were only detected in a single high-quality MAG. Without more genome representation, the significance of these observations is unclear, but as more *Nanobdellales* are discovered and grown, they may represent additional metabolic capabilities within the *Nanobdellales*. For example, a gene cluster was detected in one high-quality MAG (416-21-R1-1-10_bin.67) with two reverse ribulose monophosphate pathway genes (*hxlA, hxlB*) [67] and a phosphoribulokinase (*prk*) gene. Phosphoribulokinase is typically associated with the Calvin–Benson–Bassham (CBB) cycle for autotrophic CO_2_ fixation [68] but has been proposed as another way to produce 3-phosphoglycerate in conjunction with pentose bisphosphate pathway enzymes and RuBisCO [69]. Further, a few medium-quality MAGs from *Nanojejuna* encode a glucokinase (*glk*), which is used to convert glucose to glucose-6-phosphate for glycolysis [62].

### Putative hosts of the *Nanobdellales*

Due to the reduced genome content of all the *Nanobdellales* MAGs reported here, it is likely they only thrive in conjunction with a host for most of their metabolic needs. Without host-symbiont co-cultures or single-cell co-sorting, identifying putative hosts for the deep-sea *Nanobdellales* remains speculative. However, LGT events resulting in orthologous gene displacement between the host and symbiont have been observed in the *N. equitans-Ignicoccus hospitalis* system [70]. Similarly, other *Nanobdellati* also appear to exchange genes more frequently with their hosts than other taxa [71]. Using this rationale, we screened predicted proteins from *Nanobdellales* MAGs against the GTDB reference protein database (R10-226) to identify putative LGT events with hosts. In our analysis, we excluded any hits associated with the *Nanobdellati* (Fig. 4, Tables S9 and S10) and as an external control, we included *Ca*. Nanofervidus parviconus, whose host is the *Sulfolobales*, *Sulfurisphaera ohwakuensis* [11]. In many cases, these putative LGT events were flanked by *Nanobdellales* genes, and in some cases near-identical potential LGT genes were detected in multiple MAGs from different metagenomes (see below for examples).

**Figure 4:**
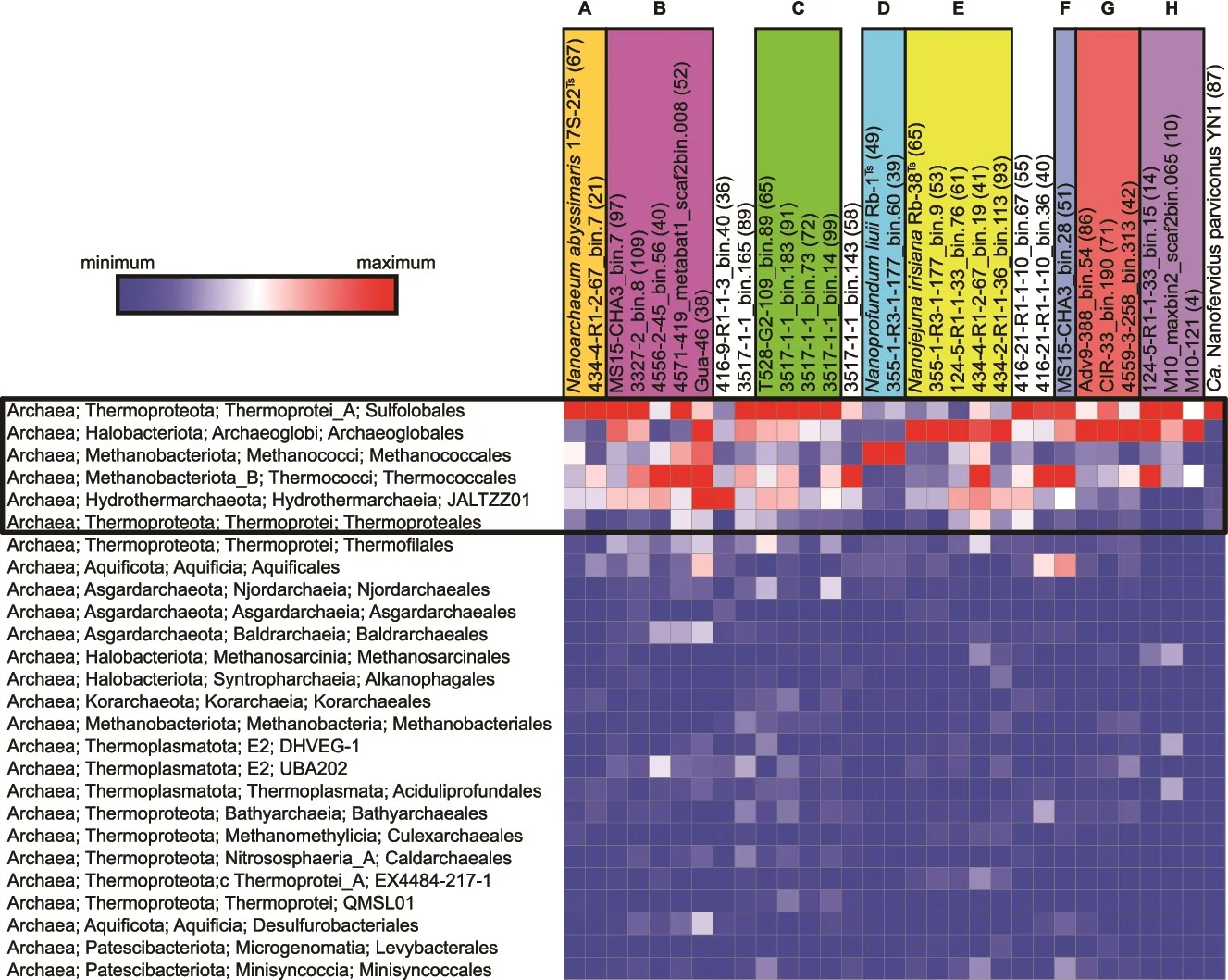
Heatmap showing putative LGT events in *Nanobdellales* MAGs and *Ca*. Nanofervidus parviconus (control). Potential LGT events are clustered at the order level using GTDB nomenclature, and only hits outside the *Nanobdellati* are considered. The colour range is displayed as a relative scale for each column, and the number of putative LGT events in each MAG is shown in parentheses.

Our approach predicted the likely host of the control, *Ca.* Nanofervidus parviconus, as a *Sulfolobales* (Fig. 4, Tables S9, S10). Further, at the genus level, *Ca.* Nanofervidus parviconus shares genes related to *Caldisphaera, Sulfurisphaera, Stygiolobus, Saccharolobus,* and *Acidianus* (Table S9). It is possible that the genes related to genera other than the host *Sulfurisphaera,* represent random LGT events, since these organisms live in such close proximity in the thermal pools. Although growth of *Ca.* Nanofervidus parviconus with *Saccharolobus* spp. was not observed in culture [11], it would nonetheless be interesting to test if some of these other genera can also act as substitute hosts. Similarly, the genome of our deep-sea MAG that is most closely related to *N. equitans*, *N. abyssimaris,* contains genes associated with members of the *Sulfolobales*, most closely related to *Ignicoccocus*, the host of *N. equitans* (Fig. 4, Tables S9, S10). Likewise, based on this analysis, the host of clade C is likely a species of *Ignicoccus*.

Our analysis also does suggest that deep-sea *Nanobdellales* may potentially associate with a broader taxonomic range of archaeal hosts. We detected a potential *Archaeoglobales* association with the deep-sea *Nanobdellales* clades E (*Nanojejuna*) and G (Fig. 4). In both *Nj. irisiana* and MAG 355-1-R3-1-177_bin.9, which were obtained from different metagenomes, a single identical *Archaeoglobales* gene was identified in a conserved six-gene *Nanojejuna* cluster (LABIEJME_00027 to LABIEJME_00032; GBEDIGFJ_00641 to GBEDIGFJ_00636). The high-quality *Archaeoglobales* MAGs associated with metagenomes in clades E and G were assigned to *Archaeoglobus, Archaeoglobus_B,* and the GTDB placeholders WAPX01 and WAKM01 [23, 27]. Since all the high-quality *Archaeoglobales* MAGs contained *dsrAB* genes, it is possible that the host and *Nj. irisiana* could be enriched under sulphate-reducing conditions. Further, based on phyloFlash analysis, *Archaeoglobales* accounted for up to 60% of archaeal diversity in some samples (Fig. S4) [25], supporting their ecological potential as hosts. Another possible host of the deep-sea *Nanobdellales* are members of the *Methanococcales,* as observed in clade D (*Nanoprofundum*, Fig. 4). In both *Np. liuii* and MAG 355-1-R3-1-177_bin.60, we detected a conserved *Nanobdellales* 32-gene cluster (FKIGOAIB_00015 to FKIGOAIB_00046; EBKDFIAH_00504 to EBKDFIAH_00535) with two putative *Methanocaldococcus* genes. These MAGs were assembled from metagenome samples collected on different research dives but from the same vent field. The *Methanococcales* accounted for about 50% of the archaeal diversity (Fig. S4) in these metagenomes. While compelling to speculate that this approach provides a glimpse into putative hosts, it could merely reflect the higher likelihood of LGT events occurring with abundant taxa.

For other clades, the predicted host relationship was less clear. For example, in clade B, while the *Sulfolobales* may be the potential host, closer inspection of the best hits did not point to a specific prevalent family within the *Sulfolobales*. In clades where we detected genes associated with several different orders, it may again suggest that LGT events have been rampant in these *Nanobdellales* lineages or illustrate the limitations of using this approach as a guide to exploring possible hosts of these deep-sea *Nanobdellales*.

In conclusion, using metagenomes from globally distributed deep-sea vent sites, we were able to greatly expand the known *Nanobdellales* from five named cultivated genera to potentially 11 new deep-sea vent genera represented by many more new species. Using the recommendations of the SeqCode, we conservatively name two new deep-sea hydrothermal *Nanobdellales* genera which more closely resemble the genomic characteristics of their terrestrial relatives, namely, *Nanojejuna irisiana* Rb-38^Ts^ gen. nov. sp. nov. and *Nanoprofundum liuii* Rb-1^Ts^ gen. nov. sp. nov. Furthermore, our data expand the known distribution of *Nanoarchaeum*, from the shallow marine to the deep sea, and we propose a new species description for the type MAG, *Nanoarchaeum abyssimaris* 17S-22^Ts^ sp. nov. Our proposal of new *Nanobdellales* from the deep sea will provide the foundation for studying the relationships of these symbionts with their potential hosts.

### Description of new taxa according to the SeqCode

#### *Nanojejuna* gen. nov.

Na.no.je.ju’na. **Gr. masc. n.**  *nanos*, a dwarf; **L. fem. adj.**  *jejuna*, hungry, fasting; **N.L. fem. n.**  *Nanojejuna*, a small hungry microorganism, referring to the predicted host-dependence of this lineage.

Members of this genus have been recovered from Rainbow vent field along the Mid-Atlantic Ridge, and from Kilo Moana vent field on the Eastern Lau Spreading Center-Valu Fa Ridge. The genus forms a monophyletic cluster in a 53-protein phylogenomic tree, and its delineation as a distinct genus is supported by average amino acid identity analysis. Based on average nucleotide identity, the MAGs represent three distinct species. The nomenclatural type of the genus is *Nanojejuna irisiana.* The SeqCode identifier for this genus is https://seqco.de/i:54898.

#### *Nanojejuna irisiana* sp. nov.

i.ri.si.a’na. **N.L. fem. adj.**  *irisiana*, of Iris, referring to the goddess of the rainbow in Greek mythology, and referencing the Rainbow vent field along the Mid-Atlantic Ridge, where the type genome for this organism was recovered.

The type genome for this organism was recovered from metagenome sequencing of a deep-sea hydrothermal vent chimney sample collected in 2008 from Rainbow vent field, Mid-Atlantic Ridge (36.229137° N, 33.903186° W), at a depth of ~2298 metres. The MAG is 613 650 bp in length, in 28 contigs, with a G + C content of 32.12%. The longest contig is 63 781 bp in length. The genome includes a 16S rRNA gene (1489 bp) and a 5S rRNA gene, 42 predicted tRNA genes, and 699 protein-coding genes. Based on the CheckM2 gradient boost model, the genome is estimated to be 91.08% complete, with 0.83% contamination. Phylogenomic reconstruction using 53 concatenated marker genes, a 16S rRNA gene phylogenetic tree, and AAI analysis places this organism in the family *Nanobdellaceae*. Based on genomic reconstruction, the species is likely host-dependent, with no recoverable ATP synthase or electron transport genes, a partial glycolytic pathway, and few genes involved in biosynthesis of amino acids, cofactors, vitamins, lipids, or nucleotides. The MAG includes several genes associated with archaella (archaeal flagella), putative S-layer genes, RNase P protein-coding genes, and an *FBPA/ase* gene potentially associated with gluconeogenesis.

The genome Rb-38^Ts^ serves as the nomenclatural type for the species, available under GenBank assembly accession number GCA_056891915.1. Raw metagenome reads are available in Sequence Read Archive (accession number SRR33214776). The SeqCode identifier for this species is https://seqco.de/i:54899.

#### *Nanoprofundum* gen. nov.

Na.no.pro.fun’dum. **Gr. masc. n.**  *nanos*, a dwarf; **L. neut. n.**  *profundum*, the depths of the sea, abyss; **N.L. neut. n.**  *Nanoprofundum*, a small microorganism from the deep ocean.

Members of this genus have been recovered in MAGs from two deep-sea hydrothermal vent chimneys from Rainbow vent field, Mid-Atlantic Ridge. This genus forms a monophyletic group in a 53-protein phylogenomic reconstruction, and its delineation as a distinct genus is supported by average amino acid identity analysis. Comparison with average nucleotide identity analysis suggests the two genomes recovered for this genus belong to a single species. The nomenclatural type of the genus is *Nanoprofundum liuii.* The SeqCode identifier for this genus is https://seqco.de/i:54897.

#### *Nanoprofundum liuii* sp. nov.

liu’i.i. **N.L. gen. n.**  *liuii*, of Liu, honouring Mr. Yitai Liu for his many contributions to the cultivation of methanogens, syntrophic, and thermophilic microorganisms.

The type genome for this species is a MAG recovered from a deep-sea hydrothermal vent chimney from Rainbow vent field, Mid-Atlantic Ridge (36.229137° N, 33.903186° W). Sample collection was performed in 2008 at approximately 2298 metres depth. The type genome consists of 39 contigs, totalling 540 544 bp in length, with a G + C content of 23.69%. The longest contig is 92 459 bp in length. The genome includes a full-length 16S rRNA gene (1500 bp), a partial 23S rRNA gene (2966 bp) and a 5S rRNA gene, 35 predicted tRNA genes, and 573 predicted protein-coding genes. The genome is estimated to be 94.79% complete by the CheckM2 gradient boost general model, with 0.24% contamination. Based on phylogenomic reconstruction and average amino acid identity analysis, this MAG clusters in the *Nanobdellaceae*. Functional genomic analysis suggests this species is likely host-dependent, with no genes recovered for the ATP synthase and very few genes involved in amino acid, vitamin, cofactor lipid, or nucleotide biosynthesis. Genes involved in the pentose bisphosphate pathway were recovered, which may provide a mechanism for degrading nucleoside monophosphates. Although the type MAG includes a few genes involved in glycolysis and gluconeogenesis, *FBPA/ase* was not identified, leaving it unclear if gluconeogenesis is functional in this species. Based on genomic data, archaella, pili, and an S-layer may be present.

The nomenclatural type for the species is Rb-1^Ts^ (Genbank accession number GCA_056891935.1). Raw reads for the metagenome are available under Sequence Read Archive accession number SRR33214776. The SeqCode identifier for this species is https://seqco.de/i:54896.

#### *Nanoarchaeum abyssimaris* sp. nov.

a.bys.si.ma’ris. **L. fem. n.**  *abyssus*, a bottomless pit, an abyss; **L. fem. n.**  *mare*, the sea; **N.L. gen. n.**  *abyssimaris*, of the deep sea.

The type genome for this species is a MAG reconstructed from metagenomic sequencing of a deep-sea hydrothermal vent chimney recovered from the Southern East Pacific Rise (17.4° S, 113.2° W). The sample was collected during a 1998/1999 expedition at approximately 2578 metres depth. The type genome is 637 833 bp in length, comprising 23 contigs, with a maximum contig length of 94 888 bp. The G + C content is 36.36%. The genome is estimated to be 93.73% to 96.59% complete using the CheckM2 neural network-specific model and gradient boost general model, respectively, with 1.04% contamination. The nomenclatural type for this species forms a monophyletic group with *Nanoarchaeum* in a phylogenomic tree generated from 53 archaeal marker genes and in a 16S rRNA gene phylogenetic tree, while average amino acid identity and average nucleotide identity analyses support its distinction as a separate species. Genomic analysis suggests the metabolic capabilities of the type MAG are similar to *Nanoarchaeum equitans*, which is obligately dependent on an archaeal host. The genome includes genes for pili and several ATP synthase subunits, but nearly all genes for glycolysis, gluconeogenesis, and biosynthesis of amino acids, lipids, vitamins, cofactors, and nucleotides were undetected.

The nomenclatural type for the species is the 17S-22^Ts^ (Genbank accession number GCA_054016815.1). Raw reads for the metagenome are available under Sequence Read Archive accession number SRR33956867. The SeqCode identifier for this species is https://seqco.de/i:54902.

## Supplementary Material

Supplementary_material_ycag208

## Data Availability

MAGs described in this study are publicly available in Genbank under accession numbers listed in Tables S2 and S3. Full phylogenetic trees used to generate Fig. 2, Fig. S2 and Fig. S3 are publicly available on iTOL at https://itol.embl.de/shared/alrlab. Predicted proteins used in this study have been deposited in the European Bioinformatics Institute (EBI) BioStudies repository under accession S-BSST3120.

## References

[1] Rinke C, Schwientek P, Sczyrba A, et al. Insights into the phylogeny and coding potential of microbial dark matter. *Nature* 2013;499:431–7. 10.1038/nature12352 23851394

[2] Göker M, Oren A. Valid publication of names of two domains and seven kingdoms of prokaryotes. *Int J Syst Evol Microbiol* 2024;74:006242. 10.1099/ijsem.0.006242 38252124

[3] Oren A, Göker M. *Candidatus* list. Lists of names of prokaryotic *Candidatus* phyla. *Int J Syst Appl Microbiol* 2023;73:005821. 10.1099/ijsem.0.005821 37159402

[4] Huang W-C, Spang A. DPANN archaea. *Curr Biol* 2025;35:R791–4. 10.1016/j.cub.2025.06.038 40829558

[5] Probst AJ, Ladd B, Jarett JK, et al. Differential depth distribution of microbial function and putative symbionts through sediment-hosted aquifers in the deep terrestrial subsurface. *Nat Microbiol* 2018;3:328–36. 10.1038/s41564-017-0098-y PMC6792436 29379208

[6] Schwank K, Bornemann TLV, Dombrowski N, et al. An archaeal symbiont-host association from the deep terrestrial subsurface. *ISME J* 2019;13:2135–9. 10.1038/S41396-019-0421-0 PMC6776059 31048756

[7] Huber H, Hohn MJ, Rachel R, et al. A new phylum of *archaea* represented by a nanosized hyperthermophilic symbiont. *Nature* 2002;417:63–7. 10.1038/417063a 11986665

[8] Wurch L, Giannone RJ, Belisle BS, et al. Genomics-informed isolation and characterization of a symbiotic *Nanoarchaeota* system from a terrestrial geothermal environment. *Nat Commun* 2016;7:12115. 10.1038/ncomms12115 PMC4935971 27378076

[9] St. John E, Liu Y, Podar M, et al. A new symbiotic nanoarchaeote (*Candidatus* Nanoclepta minutus) and its host (*Zestosphaera tikiterensis* gen. nov., sp. nov.) from a New Zealand hot spring. *Syst Appl Microbiol* 2019;42:94–106. 10.1016/j.syapm.2018.08.005 30195930

[10] Kato S, Ogasawara A, Itoh T, et al. *Nanobdella aerobiophila* gen. nov., sp. nov., a thermoacidophilic, obligate ectosymbiotic archaeon, and proposal of *Nanobdellaceae* fam. nov., *Nanobdellales* ord. nov. and *Nanobdellia* class. nov. *Int J Syst Evol Microbiol* 2022;72:005489. 10.1099/IJSEM.0.005489 35993221

[11] Johnson MD, Sakai HD, Paul B, et al. Large attachment organelle mediates interaction between *Nanobdellota* archaeon YN1 and its host. *ISME J* 2024;18:wrae154. 10.1093/ismejo/wrae154 PMC11420986 39113594

[12] Sakai HD, Nur N, Kato S, et al. Insight into the symbiotic lifestyle of DPANN archaea revealed by cultivation and genome analyses. *Proc Natl Acad Sci U S A* 2022;119:e2115449119. 10.1073/pnas.2115449119 PMC8784108 35022241

[13] Golyshina OV, Toshchakov SV, Makarova KS, et al. ‘ARMAN’ archaea depend on association with euryarchaeal host in culture and in situ. *Nat Commun* 2017;8:60. 10.1038/s41467-017-00104-7 PMC5498576 28680072

[14] Hamm JN, Erdmann S, Eloe-Fadrosh EA, et al. Unexpected host dependency of Antarctic *Nanohaloarchaeota*. *Proc Natl Acad Sci U S A* 2019;116:14661–70. 10.1073/pnas.1905179116 PMC6642349 31253704

[15] Reva O, Messina E, La Cono V, et al. Functional diversity of nanohaloarchaea within xylan-degrading consortia. *Front Microbiol* 2023;14:1182464. 10.3389/fmicb.2023.1182464 PMC10266531 37323909

[16] La Cono V, Messina E, Rohde M, et al. Symbiosis between nanohaloarchaeon and haloarchaeon is based on utilization of different polysaccharides. *Proc Natl Acad Sci U S A* 2020;117:20223–34. 10.1073/pnas.2007232117 PMC7443923 32759215

[17] Waters E, Hohn MJ, Ahel I, et al. The genome of *Nanoarchaeum equitans*: insights into early archaeal evolution and derived parasitism. *Proc Natl Acad Sci U S A* 2003;100:12984–8. 10.1073/pnas.1735403100 PMC240731 14566062

[18] Mohanty S, Jobichen C, Chichili VPR, et al. Structural basis for a unique ATP synthase core complex from *Nanoarcheaum equitans*. *J Biol Chem* 2015;290:27280–96. 10.1074/jbc.M115.677492 PMC4646375 26370083

[19] Flores GE, Shakya M, Meneghin J, et al. Inter-field variability in the microbial communities of hydrothermal vent deposits from a back-arc basin. *Geobiology* 2012;10:333–46. 10.1111/j.1472-4669.2012.00325.x 22443386

[20] McCliment EA, Voglesonger KM, O’Day PA, et al. Colonization of nascent, deep-sea hydrothermal vents by a novel Archaeal and Nanoarchaeal assemblage. *Environ Microbiol* 2006;8:114–25. 10.1111/j.1462-2920.2005.00874.x 16343327

[21] Zhou Z, St. John E, Anantharaman K, et al. Global patterns of diversity and metabolism of microbial communities in deep-sea hydrothermal vent deposits. *Microbiome* 2022;10:241. 10.1186/s40168-022-01424-7 PMC9793634 36572924

[22] St. John E, Flores GE, Meneghin J, et al. Deep-sea hydrothermal vent metagenome-assembled genomes provide insight into the phylum *Nanoarchaeota*. *Environ Microbiol Rep* 2019;11:262–70. 10.1111/1758-2229.12740 30768760

[23] St. John E, Reysenbach A-L. Global deep-sea hydrothermal deposit metagenomes and metagenome-assembled genomes over time and space. *Sci Data* 2026;13:283. 10.1038/s41597-026-06612-w PMC12920610 41540059

[24] Reysenbach A-L, St. John E, Meneghin J, et al. Complex subsurface hydrothermal fluid mixing at a submarine arc volcano supports distinct and highly diverse microbial communities. *Proc Natl Acad Sci U S A* 2020;117:32627–38. 10.1073/pnas.2019021117 PMC7768687 33277434

[25] Gruber-Vodicka HR, Seah BKB, Pruesse E. phyloFlash: rapid small-subunit rRNA profiling and targeted assembly from metagenomes. *mSystems* 2020;5:e00920–0. 10.1128/msystems.00920-20 PMC7593591 33109753

[26] Chaumeil P-A, Mussig AJ, Hugnholtz P, et al. GTDB-Tk: a toolkit to classify genomes with the genome taxonomy database. *Bioinformatics* 2020;36:1925–7. 10.1093/BIOINFORMATICS/BTZ848 PMC7703759 31730192

[27] Parks DH, Chaumeil P-A, Mussig AJ, et al. GTDB release 10: a complete and systematic taxonomy for 715 230 bacterial and 17 245 archaeal genomes. *Nucleic Acids Res* 2026;54:D743–54. 10.1093/nar/gkaf1040 PMC12807784 41123020

[28] von Meijenfeldt FAB, Arkhipova K, Cambuy DD, et al. Robust taxonomic classification of uncharted microbial sequences and bins with CAT and BAT. *Genome Biol* 2019;20:217. 10.1186/s13059-019-1817-x PMC6805573 31640809

[29] Qi Y-L, Zhang H-T, Li M, et al. Recovery of nearly 3,000 archaeal genomes from 152 terrestrial geothermal spring metagenomes. *Sci Data* 2025;12:151. 10.1038/s41597-025-04493-z PMC11770067 39865091

[30] Rocha U, Kasmanas JC, Kallies R, et al. MuDoGeR: multi-domain genome recovery from metagenomes made easy. *Mol Ecol Resour* 2024;24:e13904. 10.1111/1755-0998.13904 37994269

[31] Chklovski A, Parks DH, Woodcroft BJ, et al. CheckM2: a rapid, scalable and accurate tool for assessing microbial genome quality using machine learning. *Nat Methods* 2023;20:1203–12. 10.1038/s41592-023-01940-w 37500759

[32] Hyatt D, Chen G-L, LoCascio PF, et al. Prodigal: prokaryotic gene recognition and translation initiation site identification. *BMC Bioinformatics* 2010;11:119. 10.1186/1471-2105-11-119 PMC2848648 20211023

[33] Seemann T . Prokka: rapid prokaryotic genome annotation. *Bioinformatics* 2014;30:2068–9. 10.1093/bioinformatics/btu153 24642063

[34] Chan PP, Lin BY, Mak AJ, et al. tRNAscan-SE 2.0: improved detection and functional classification of transfer RNA genes. *Nucleic Acids Res* 2021;49:9077–96. 10.1093/nar/gkab688 PMC8450103 34417604

[35] Randau L, Pearson M, Söll D. The complete set of tRNA species in *Nanoarchaeum equitans*. *FEBS Lett* 2005;579:2945–7. 10.1016/j.febslet.2005.04.051 15893316

[36] Couvin D, Bernheim A, Toffano-Nioche C, et al. CRISPRCasFinder, an update of CRISRFinder, includes a portable version, enhanced performance and integrates search for Cas proteins. *Nucleic Acids Res* 2018;46:W246–51. 10.1093/nar/gky425 PMC6030898 29790974

[37] Johnson M, Zaretskaya I, Raytselis Y, et al. NCBI BLAST: a better web interface. *Nucleic Acids Res* 2008;36:W5–9. 10.1093/nar/gkn201 PMC2447716 18440982

[38] Altschul SF, Gish W, Miller W, et al. Basic local alignment search tool. *J Mol Biol* 1990;215:403–10. 10.1016/S0022-2836(05)80360-2 2231712

[39] Sayers EW, Beck J, Bolton EE, et al. Database resources of the National Center for biotechnology information in 2025. *Nucleic Acids Res* 2025;53:D20–9. 10.1093/nar/gkae979 PMC11701734 39526373

[40] Pritchard L, Glover RH, Humphris S, et al. Genomics and taxonomy in diagnostics for food security: soft-rotting enterobacterial plant pathogens. *Anal Methods* 2016;8:12–24. 10.1039/C5AY02550H

[41] Yoon S-H, Ha S-M, Kwon S, et al. Introducing EzBioCloud: a taxonomically united database of 16S rRNA gene sequences and whole-genome assemblies. *Int J Syst Evol Microbiol* 2017;67:1613–7. 10.1099/ijsem.0.001755 PMC5563544 28005526

[42] Chuvochina M, Gerken J, Frentrup M, et al. SILVA in 2026: a global core biodata resource for rRNA within the DSMZ digital diversity. *Nucleic Acids Res* 2026;54:D334–41. 10.1093/NAR/GKAF1247 PMC12807666 41251139

[43] Pruesse E, Peplies J, Glöckner FO. SINA: accurate high-throughput multiple sequence alignment of ribosomal RNA genes. *Bioinformatics* 2012;28:1823–9. 10.1093/BIOINFORMATICS/BTS252 PMC3389763 22556368

[44] Stamatakis A . RAxML version 8: a tool for phylogenetic analysis and post-analysis of large phylogenies. *Bioinformatics* 2014;30:1312–3. 10.1093/bioinformatics/btu033 PMC3998144 24451623

[45] Wang Q, Cole JR. Updated RDP taxonomy and RDP classifier for more accurate taxonomic classification. *Microbiol Resour Announc* 2024;13:e01063–23. 10.1128/MRA.01063-23 PMC11008197 38436268

[46] Price MN, Dehal PS, Arkin AP. FastTree 2 – approximately maximum-likelihood trees for large alignments. *PloS One* 2010;5:e9490. 10.1371/journal.pone.0009490 PMC2835736 20224823

[47] Shimodaira H, Hasegawa M. Multiple comparisons of log-likelihoods with applications to phylogenetic inference. *Mol Biol Evol* 1999;16:1114–6. 10.1093/OXFORDJOURNALS.MOLBEV.A026201

[48] Letunic I, Bork P. Interactive tree of life (iTOL) v6: recent updates to the phylogenetic tree display and annotation tool. *Nucleic Acids Res* 2024;52:W78–82. 10.1093/NAR/GKAE268 PMC11223838 38613393

[49] Hedlund BP, Chuvochina M, Hugenholtz P, et al. SeqCode: a nomenclatural code for prokaryotes described from sequence data. *Nat Microbiol* 2022;7:1702–8. 10.1038/s41564-022-01214-9 PMC9519449 36123442

[50] Kanehisa M, Sato Y, Morishima K. BlastKOALA and GhostKOALA: KEGG tools for functional characterization of genome and metagenome sequences. *J Mol Biol* 2016;428:726–31. 10.1016/j.jmb.2015.11.006 26585406

[51] Makarova KS, Wolf YI, Koonin EV. Archaeal clusters of orthologous genes (arCOGs): an update and application for analysis of shared features between *Thermococcales*, *Methanococcales*, and *Methanobacteriales*. *Life* 2015;5:818–40. 10.3390/life5010818 PMC4390880 25764277

[52] Marchler-Bauer A, Bryant SH. CD-search: protein domain annotations on the fly. *Nucleic Acids Res* 2004;32:W327–31. 10.1093/nar/gkh454 PMC441592 15215404

[53] Buchfink B, Xie C, Huson DH. Fast and sensitive protein alignment using DIAMOND. *Nat Methods* 2015;12:59–60. 10.1038/nmeth.3176 25402007

[54] Coffin MF, Gahagan LM, Lawver LA. Present-day plate boundary digital data compilation. *University of Texas Institute for Geophysics Technical Report* 1997;174:5.

[55] Urayama S-I, Fukudome A, Hirai M, et al. Double-stranded RNA sequencing reveals distinct riboviruses associated with thermoacidophilic bacteria from hot springs in Japan. *Nat Microbiol* 2024;9:514–23. 10.1038/S41564-023-01579-5 PMC10847044 38233646

[56] Fricke H, Giere O, Stetter K, et al. Hydrothermal vent communities at the shallow subpolar Mid-Atlantic ridge. *Mar Biol* 1989;102:425–9. 10.1007/BF00428495

[57] Anderson RE, Reveillaud J, Reddington E, et al. Genomic variation in microbial populations inhabiting the marine subseafloor at deep-sea hydrothermal vents. *Nat Commun* 2017;8:1114. 10.1038/s41467-017-01228-6 PMC5655027 29066755

[58] Konstantinidis KT, Rosselló-Móra R, Amann R. Uncultivated microbes in need of their own taxonomy. *ISME J* 2017;11:2399–406. 10.1038/ismej.2017.113 PMC5649169 28731467

[59] Randau L . RNA processing in the minimal organism *Nanoarchaeum equitans*. *Genome Biol* 2012;13:R63. 10.1186/gb-2012-13-7-r63 PMC3491384 22809431

[60] Gaisin VA, van Wolferen M, Albers S-J, et al. Distinct life cycle stages of an ectosymbiotic DPANN archaeon. *ISME J* 2024;18:wrae076. 10.1093/ismejo/wrae076 PMC11104419 38691426

[61] Randau L, Schröder I, Söll D. Life without RNase P. *Nature* 2008;453:120–3. 10.1038/nature06833 18451863

[62] Bräsen C, Esser D, Rauch B, et al. Carbohydrate metabolism in Archaea: current insights into unusual enzymes and pathways and their regulation. *Microbiol Mol Biol Rev* 2014;78:89–175. 10.1128/mmbr.00041-13 PMC3957730 24600042

[63] Aono R, Sato T, Yano A, et al. Enzymatic characterization of AMP phosphorylase and ribose-1,5-bisphosphate isomerase functioning in an archaeal AMP metabolic pathway. *J Bacteriol* 2012;194:6847–55. 10.1128/jb.01335-12 PMC3510611 23065974

[64] Aono R, Sato T, Imanaka T, et al. A pentose bisphosphate pathway for nucleoside degradation in *Archaea*. *Nat Chem Biol* 2015;11:355–60. 10.1038/nchembio.1786 25822915

[65] Sato T, Atomi H, Imanaka T. Archaeal type III RuBisCOs function in a pathway for AMP metabolism. *Science* 2007;315:1003–6. 10.1126/science.1135999 17303759

[66] Youssef NH, Rinke C, Stepanauskas R, et al. Insights into the metabolism, lifestyle and putative evolutionary history of the novel archaeal phylum ‘*Diapherotrites’*. *ISME J* 2015;9:447–60. 10.1038/ismej.2014.141 PMC4303637 25083931

[67] Kato N, Yurimoto H, Thauer RK. The physiological role of the ribulose monophosphate pathway in *Bacteria* and *Archaea*. *Biosci Biotechnol Biochem* 2006;70:10–21. 10.1271/bbb.70.10 16428816

[68] Garritano AN, Song W, Thomas T. Carbon fixation pathways across the bacterial and archaeal tree of life. *PNAS Nexus* 2022;1:pgac226. 10.1093/pnasnexus/pgac226 PMC9802188 36712370

[69] Kono T, Mehrotra S, Endo C, et al. A RuBisCO-mediated carbon metabolic pathway in methanogenic archaea. *Nat Commun* 2017;8:14007. 10.1038/ncomms14007 PMC5241800 28082747

[70] Podar M, Anderson I, Makarova KS, et al. A genomic analysis of the archaeal system *Ignicoccus hospitalis*-*Nanoarchaeum equitans*. *Genome Biol* 2008;9:R158. 10.1186/gb-2008-9-11-r158 PMC2614490 19000309

[71] Dombrowski N, Williams TA, Sun J, et al. *Undinarchaeota* illuminate DPANN phylogeny and the impact of gene transfer on archaeal evolution. *Nat Commun* 2020;11:3939. 10.1038/s41467-020-17408-w PMC7414124 32770105

