# Supplementary material for: Expanding the global diversity of Nanobdellales from deep-sea hydrothermal vents
Source: ISME Commun. 2026 Oct 1;6(1):ycag280. doi: 10.1093/ismeco/ycag280 (PMC13718433; doi:10.1093/ismeco/ycag280)
Supplement: Supplementary file 1 [file supplementary_material_ycag208.zip › supplementary_material_29Sept_submit_ycag280.pdf]

Supplementary material for

Expanding the global diversity of *Nanobdellales* from deep-sea hydrothermal vents

Anna-Louise Reysenbach[1]*, Emily St. John[1] and Ryan Catchpole[2]

Supplementary Material

This file includes one supplementary table (S2) and four supplementary figures (S1 through S4). Nine additional supplementary tables (S1, S3 through S10) are included separately as an Excel workbook.

Table S2. Genome features of all the deep-sea vent *Nanobdellales* MAGs in this study. Type genomes for novel genera and species proposed in this study are shown in bold.

MAG	Clade	Assembly accession	Ref	Site	Comp gen (%)	Comp spec (%)	Con (%)	Genome size (bp)	GC (%)	Contigs (#)	Prot (#)	16S rRNA gene (bp)
***Nanoarchaeum abyssimaris* 17S-22^{Ts}**	A	GCA_054016815.1	[1]	17º 25' S, SEPR	96.59	93.73	1.04	637 833	36.36	23	723	1497
434-4-R1-2-67_bin.7	A	GCA_056891545.1	[1]	Kilo Moana, ELSC-VFR	75.49	58.77	3.78	463 095	31.67	120	513	--
3327-2_bin.8	B	GCA_056892115.1	[1]	17º 25' S, SEPR	88.31	83.36	0.72	640 590	30.6	77	796	--
4556-2-45_bin.56	B	GCA_056891485.1	[1]	Guaymas Basin	69.64	54.24	1.29	363 863	30.78	93	410	--
4571-419_metabat1_scaf2bin.008	B	GCA_027062685.1	[2]	TC, Guaymas Basin	73.36	64.28	0.99	455 705	30.62	121	534	779
Gua-46	B	GCA_004285905.1	[3]	TC, Guaymas Basin	67.26	54.11	0.89	356 822	31.06	100	396	779
MS15-CHA3_bin.7	B	GCA_054017035.1	[1]	Snake Pit, MAR	94.84	90.1	2.96	648 387	30.09	22	812	--
416-9-R1-1-3_bin.40	S	GCA_056891755.1	[1]	Maka, NELSC	63.28	38.35	1.15	291 411	32.19	87	358	--
3517-1-1_bin.165	S	GCA_056892135.1	[1]	Guaymas Basin	83.47	73.52	0.34	621 288	31.48	11	710	--
3517-1-1_bin.14	C	GCA_056892095.1	[1]	Guaymas Basin	90.23	87.23	2.63	689 609	31.01	55	808	--
3517-1-1_bin.183	C	GCA_056892015.1	[1]	Guaymas Basin	91.54	87.91	6.8	686 324	32.52	41	760	509
3517-1-1_bin.73	C	GCA_056891995.1	[1]	Guaymas Basin	81.17	67.82	9.39	566 303	32.52	33	679	806
T528-G2-109_bin.89	C	GCA_056891225.1	[1]	Guaymas Basin	77.64	66.94	0.79	482 148	28.29	75	545	--
3517-1-1_bin.143	S	GCA_053994055.1	[1]	Guaymas Basin	90.86	89.54	0.26	641 398	36.15	33	743	551
***Nanoprofundum liuii* Rb-1^{Ts}**	D	GCA_056891935.1	[1]	Rainbow, MAR	94.79	78.76	0.24	540 544	23.69	39	573	1500
355-1-R3-1-177_bin.60	D	GCA_056891895.1	[1]	Rainbow, MAR	91.99	71.82	0.29	502 194	23.57	35	542	1500
***Nanojejuna irisiana* Rb-38^{Ts}**	E	GCA_056891915.1	[1]	Rainbow, MAR	91.08	80.73	0.83	613 650	32.12	28	699	1489
355-1-R3-1-177_bin.9	E	GCA_056891815.1	[1]	Rainbow, MAR	88.52	78.5	0.26	543 675	32.34	79	617	1244
124-5-R1-1-33_bin.76	E	GCA_056891365.1	[1]	Kilo Moana, ELSC-VFR	87.63	78.61	0.86	616 090	28.92	41	689	1481
434-2-R1-1-36_bin.113	E	GCA_056891605.1	[1]	Kilo Moana, ELSC-VFR	70.88	61.37	0.28	705 379	29.4	126	784	--
434-4-R1-2-67_bin.19	E	GCA_056891585.1	[1]	Kilo Moana, ELSC-VFR	73.83	54.46	0.62	421 106	29.52	116	459	--
416-21-R1-1-10_bin.67	S	GCA_056891785.1	[1]	Maka, NELSC	93.86	88.64	0.68	737 933	28.42	5	811	404
416-21-R1-1-10_bin.36	S	GCA_056891735.1	[1]	Maka, NELSC	93.66	86.56	0.33	616 283	30.11	15	698	404
MS15-CHA3_bin.28	F	GCA_056891305.1	[1]	Snake Pit, MAR	90.39	88.5	0.33	624 038	30.23	34	729	757

MC-1	F	GCA_020697515.1	[3]	Mid-Cayman Rise	84.66	76.93	0.24	469 257	32.21	55	499	1517
4559-3-258_bin.313	G	GCA_056891465.1	[1]	Guaymas Basin	83.68	75.31	1.57	521 119	35.28	88	593	1478
Adv9-388_bin.54	G	GCA_056891445.1	[1]	9ºN, EPR	89.65	87.64	0.73	6799 99	30.41	31	772	515
CIR-33_bin.190	G	GCA_056891345.1	[1]	Kairei, CIR	79.74	72.8	1.54	595 203	32.68	54	672	--
124-5-R1-1-33_bin.15	H	GCA_056892175.1	[1]	Kilo Moana, ELSC-VFR	90.95	86.32	0.68	544 244	31.39	21	617	--
M10-121	H	GCA_004285895.1	[3]	Mariner, ELSC-VFR	54.14	35.53	0.75	214 648	32.21	87	210	--
M10_maxbin2_scaf2bin.065	H	GCA_027035585.1	[2]	Mariner, ELSC-VFR	66.25	54.89	0.41	362 429	31.8	149	398	--

CheckM2 was used to estimate completeness and contamination and % G+C content was determined with the get_gc_by_bin.pl script (https://github.com/ALR-Lab/get_gc_by_bin.pl). Clade designations are shown in Figure 2, and singletons are marked as “S”. Novel taxa proposed under the SeqCode are shown with the superscript “Ts”. Ref = reference; Comp gen (%) = estimated completeness under the CheckM2 general model as a percentage; Comp spec (%) = percent CheckM2 estimated completeness under the specific model; Con (%) = percent estimated contamination; GC (%) = G+C content as a percentage; Contigs (#) = number of contigs in each MAG; Prot (#) = number of predicted proteins per MAG; 16S rRNA gene (bp) = length of predicted 16S rRNA gene(s) in base pairs; EPR = East Pacific Rise; ELSC-VFR = Eastern Lau Spreading Center-Valu Fa Ridge; MAR = Mid-Atlantic Ridge; NELSC = Northeastern Lau Spreading Center; NZ = New Zealand; SEPR = Southern East Pacific Rise; TC = thermocouple array.

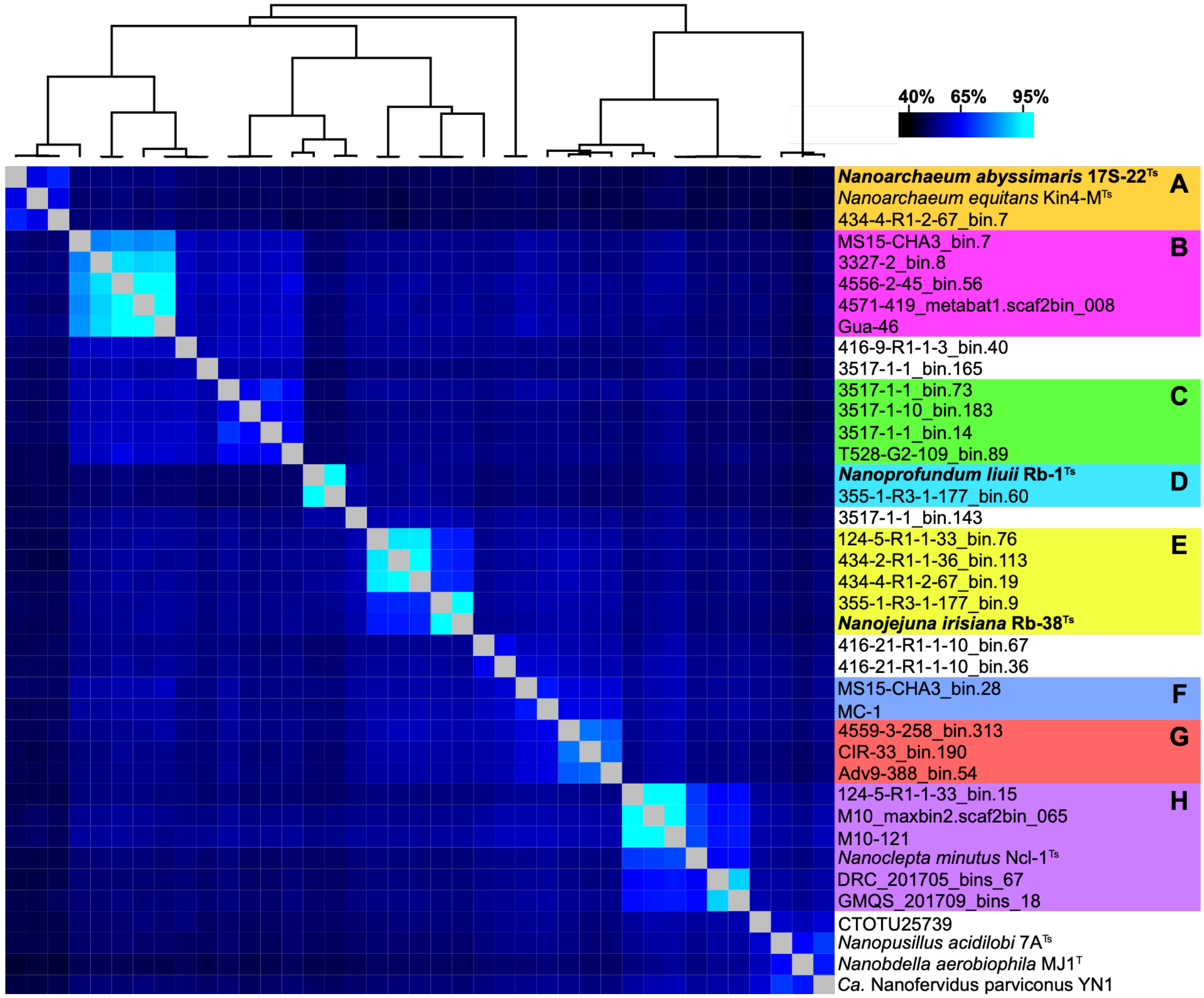

Figure S1. Average amino acid identity analysis of *Nanobdellales* MAGs and reference genomes, with color indicating AAI values between sequences. Novel taxa proposed in this study are shown in bold text, and coloured boxes indicate genus-level clades shown in Figure 2. Hierarchical clustering is displayed at the top, generated using one minus Pearson correlation (average linkage). Nomenclatural types proposed under the SeqCode are shown with a superscript "Ts", and the type strain *Nanobdella aerobiophila* is shown with a superscript "T".

Alt text: Graphical depiction of AAI values, showing clustering of putative novel genera. Higher (hotter) values indicate more closely related lineages.

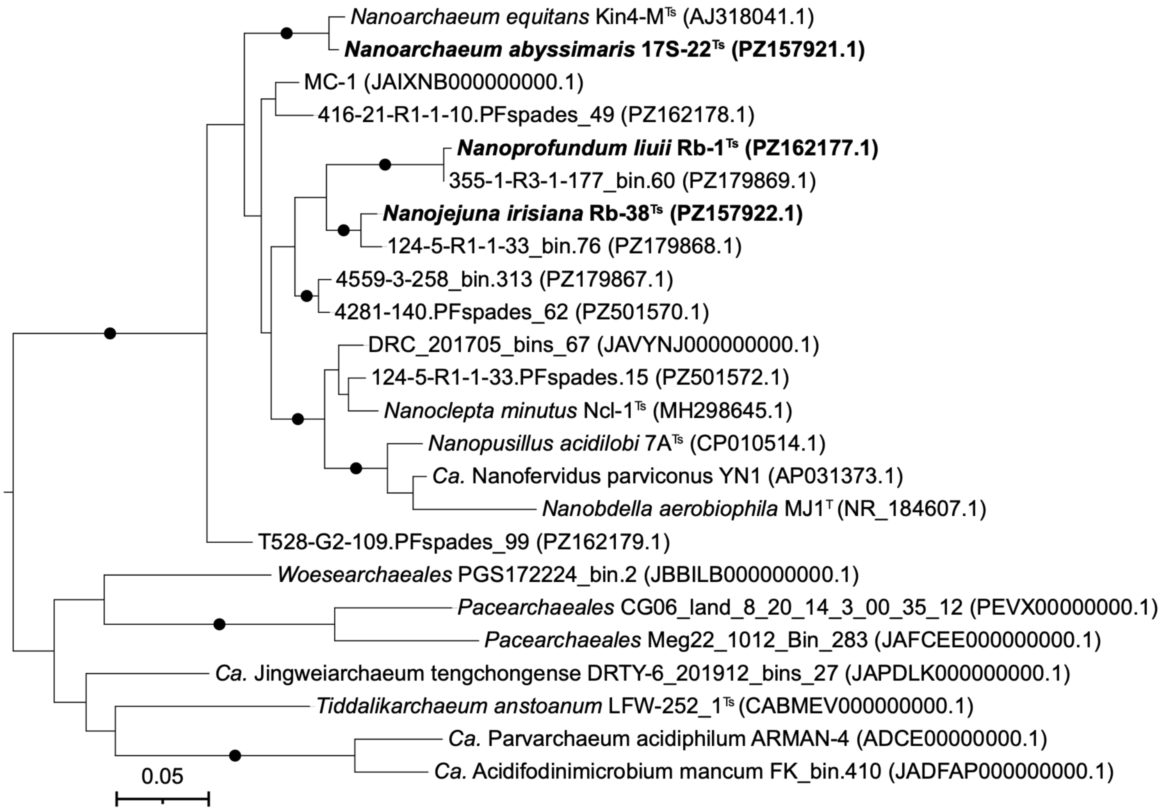

Figure S2. Maximum likelihood phylogenetic tree of the 16S rRNA gene showing the placement of novel *Nanobdellales* in relation to their cultivated relatives. MAG-derived 16S rRNA genes and sequences assembled with phyloFlash (identifier "PFspades") are shown. The 355-1-R3-1-177_bin.9 16S rRNA gene sequence is not included as it was identical to the *Nj. irisiana* Rb-38^{Ts} sequence across their shared length. Type strains are indicated with the superscript "T" and type sequences proposed under the SeqCode are shown with the superscript "Ts". Bootstrap support from 80 to 100% is shown as filled circles, and the scale bar indicates 0.05 substitutions per nucleotide. The displayed tree was pruned from a larger archaeal alignment rooted with *Escherichia coli* (NR_024570.1), *Bacillus subtilis* (NR_112116.2), *Deinococcus radiodurans* (NR_026401.1) and *Flexibacter flexilis* (NR_044739.2). The full tree used to generate this figure is available on iTOL at https://itol.embl.de/shared/alrlab.

Alt text. Phylogenetic tree of 16S rRNA genes from novel deep-sea *Nanobdellales,* and their relatives. Newly proposed lineages *Nanoprofundum, Nanojejuna* and *Nanoarchaeum abyssimaris* are included, and other members of the *Nanobdellati* are shown for context.

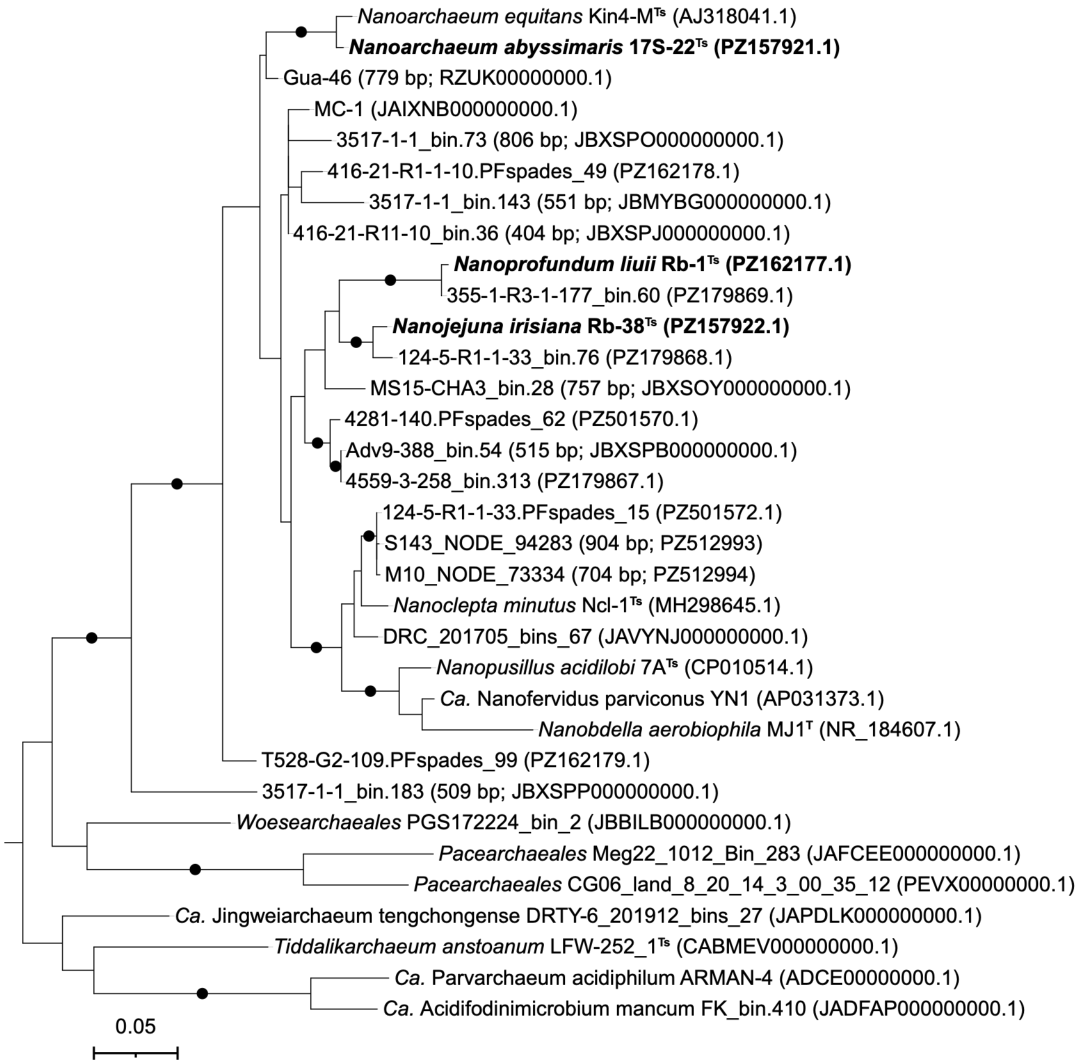

Figure S3. Maximum likelihood phylogenetic tree of 16S rRNA gene sequences, including gene fragments recovered from MAGs, phyloFlash reconstruction (identifier "PFspades") and the M10 and S143 assemblies. Small subunit rRNA genes from 355-1-R3-1-177_bin.9 and 4571-419_metabat1_scaf2bin.008 are not shown as these shared 100% sequence identity with *Nj. irisiana* Rb-38^{Ts} and Gua-46, respectively. The 416-21-R1-1-10.bin_67 16S rRNA gene fragment was also removed as it was identical to 416-21-R1-1-10.bin_36 after SILVA alignment filtering. Novel genera and species proposed in this study are in bold text. The superscript "T" indicates a type strain and the superscript "Ts" indicates a proposed type genome under the SeqCode. Genbank accessions are shown in parentheses, and gene length for fragments is indicated. Bootstrap support (≥80%) is shown with filled black circles, and the scale bar represents 0.05 substitutions per nucleotide. The tree was pruned and rooted as described for Figure S2, and the full tree used to generate this figure is available on iTOL at https://itol.embl.de/shared/alrlab.

Alt text. Phylogenetic reconstruction of *Nanobdellales* 16S rRNA genes, including 16S rRNA gene fragments recovered from deep-sea MAGs.

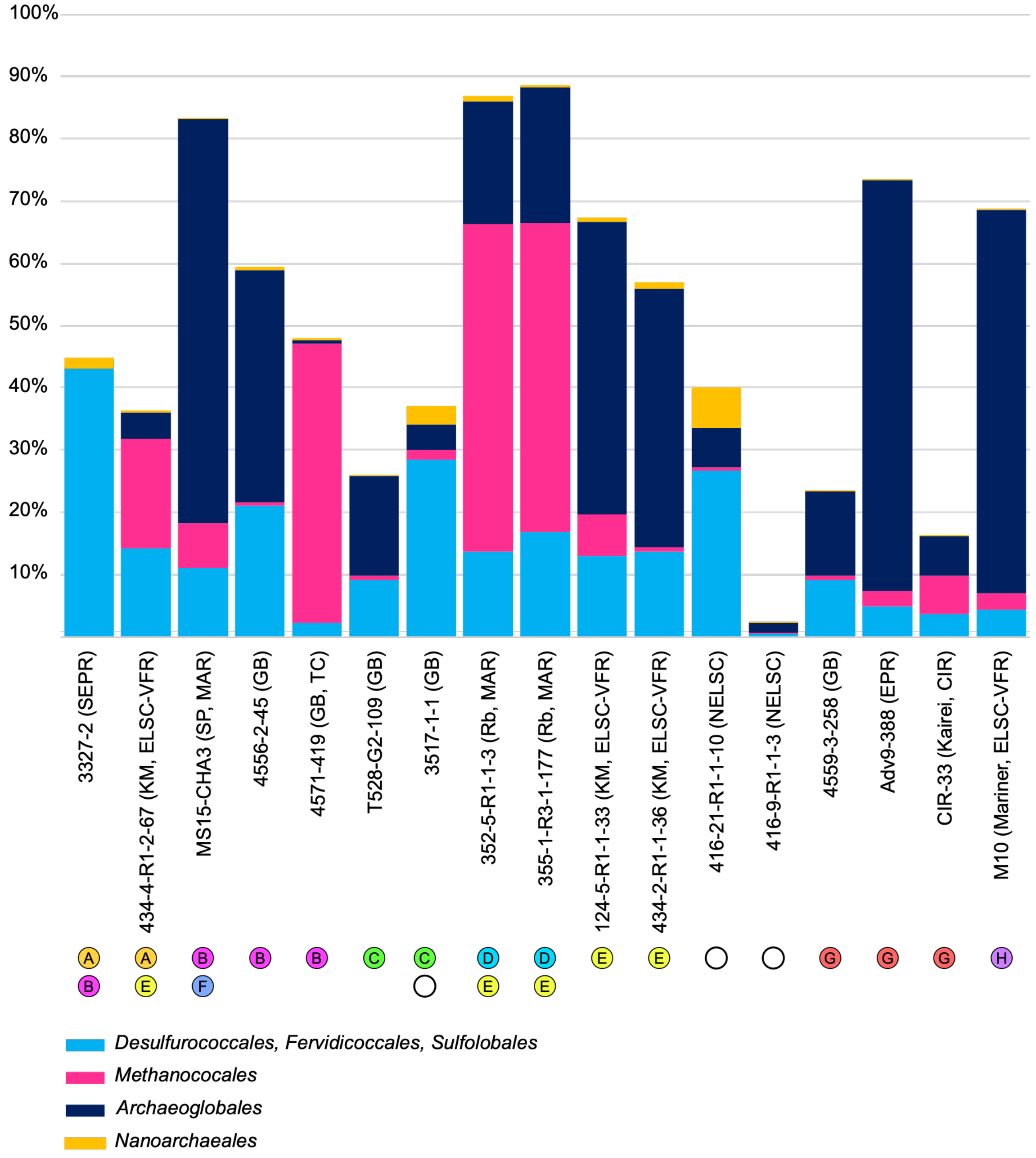

Figure S4. Relative abundance of archaeal 16S rRNA gene fragments assigned to the *Nanoarchaeales* and to potential host lineages with phyloFlash [4], shown as a percentage. Nomenclature is displayed using SILVA taxonomy [5]. Deep-sea vent MAG clades from each sample are displayed as coloured circles corresponding to Figure 2, and singleton MAGs are represented with open circles. CIR = Central Indian Ridge; ELSC-VFR = Eastern Lau Spreading Center-Valu Fa Ridge; EPR = East Pacific Rise; GB = Guaymas Basin; KM = Kilo Moana; MAR = Mid-Atlantic Ridge; NELSC = Northeastern Lau Spreading Center; Rb = Rainbow; SEPR = Southern East Pacific Rise; SP = Snake Pit; TC = thermocouple array.

Alt text. Bar chart showing abundance of *Nanobdellales* and potential host lineages in deep-sea vent metagenomes. Putative host taxa include the *Methanococcales, Archaeoglobales* and a group comprising *Desulfurococcales, Fervidicoccales* and *Sulfolobales*.

Supplementary References

1. St. John E, Reysenbach A-L. Global deep-sea hydrothermal deposit metagenomes and metagenome-assembled genomes over time and space. *Sci Data* 2026;**13**:283. https://doi.org/10.1038/s41597-026-06612-w

2. Zhou Z, St. John E, Anantharaman K, Reysenbach A-L. Global patterns of diversity and metabolism of microbial communities in deep-sea hydrothermal vent deposits. *Microbiome* 2022;**10**:241. https://doi.org/10.1186/s40168-022-01424-7

3. St. John E, Flores GE, Meneghin J, Reysenbach A-L. Deep-sea hydrothermal vent metagenome-assembled genomes provide insight into the phylum *Nanoarchaeota*. *Environ Microbiol Rep* 2019;**11**:262–70. https://doi.org/10.1111/1758-2229.12740

4. Gruber-Vodicka HR, Seah BKB, Pruesse E. phyloFlash: rapid small-subunit rRNA profiling and targeted assembly from metagenomes. *mSystems* 2020;**5**:e00920-20. https://doi.org/10.1128/msystems.00920-20

5. Quast C, Pruesse E, Yilmaz P, Gerken J, Schweer T *et al*. The SILVA ribosomal RNA gene database project: improved data processing and web-based tools. *Nucleic Acids Res* 2013;**41**:D590–6. https://doi.org/10.1093/nar/gks1219
